# Supplementary material for: Analysis of PPARγ Signaling Activity in Psoriasis
Source: Int J Mol Sci. 2021 Aug 10;22(16):8603. doi: 10.3390/ijms22168603 (PMC8395241; doi:10.3390/ijms22168603)
Supplement: Supplementary file 1 [file ijms-22-08603-s001.zip › Supplemental materials_Analysis of PPARg signaling activity in psoriasis/Pathway models/Models images and html files/Differentiation of psoriatic T cells/6326.html]

IL4


# Protein IL4

|  |  |
| --- | --- |
| URN | urn:agi-llid:3565 |
| Total Entities | 0 |
| Connectivity | 9899 |
| Name | IL4 |
| Description | interleukin 4 |
| Notes | The protein encoded by this gene is a pleiotropic cytokine produced by activated T cells. This cytokine is a ligand for interleukin 4 receptor. The interleukin 4 receptor also binds to IL13, which may contribute to many overlapping functions of this cytokine and IL13. STAT6, a signal transducer and activator of transcription, has been shown to play a central role in mediating the immune regulatory signal of this cytokine. This gene, IL3, IL5, IL13, and CSF2 form a cytokine gene cluster on chromosome 5q, with this gene particularly close to IL13. This gene, IL13 and IL5 are found to be regulated coordinately by several long-range regulatory elements in an over 120 kilobase range on the chromosome. Two alternatively spliced transcript variants of this gene encoding distinct isoforms have been reported. [provided by RefSeq, Jul 2008] |
| Primary Cell Localization | Extracellular |
| Class | Ligand |
| ObjectType | Protein |

---

|  |  |
| --- | --- |
| Pathway | T-Cell Dependent B-Cell Activation |
|  | Immunoglobulin Class-Switch Recombination Activation |
|  | T-Cell Maturation (Hypothesis) |
|  | CD8+ T-Cell Activation |
|  | Th2-Cell Differentiation |
|  | T-Cell Receptor Signaling |
|  | Lipoxins and Resolvins in Inflammation Resolution |
|  | Keep out Signal: LTF Inhibits Neutrophil Recruitment and Inflammation |
|  | Histamine Effects in Inflammation |
|  | Basophil Activation |
|  | Eosinophil Activation |
|  | Mast-Cell Activation without Degranulation through CYSLTR1/CYSLTR2 Signaling |
|  | Mast-Cells De Novo Synthesized Mediators via IgE Independent Signaling |
|  | anti-Inflammatory Function of Macrophage M2 Lineage |
|  | Neutrophil Recruitment and Priming |
|  | Nociception Expression Targets Signaling |
|  | CNR1/2 -> IL1B/2/4/6/10 Production |
|  | Hematopoietic Cell Lineage: B-cell (mouse) |
|  | Proteins Involved in Atherosclerosis |
|  | Proteins Involved in Myocarditis |
|  | Atopic Dermatitis Onset |
|  | Mast-Cells Activation in Atopic Dermatitis |
|  | Genes with Mutations Associated with Atopic Dermatitis |
|  | Acute Phase in Atopic Dermatitis |
|  | Proteins Involved in Atopic Dermatitis |
|  | Atopic Dermatitis |
|  | Proteins Involved in Psoriasis |
|  | Genes with Mutations Associated with Psoriasis |
|  | T-Cells Differentiation Block in Psoriasis |
|  | Proteins with Altered Expression in Psoriasis |
|  | Proteins Involved in Systemic Lupus Erythematosus |
|  | Th1-Cell Function in Systemic Lupus Erythematosus |
|  | Th2-Cell Function in Systemic Lupus Erythematosus |
|  | Genes with Mutations Associated with Vitiligo |
|  | Vitiligo |
|  | T-Cell Cytotoxic Response against Melanocytes in Vitiligo |
|  | Humoral Immunity in Vitiligo |
|  | Natural Killer T-Cell Roles in Diabetes Mellitus Type 1 |
|  | Proteins Involved in Diabetes Mellitus Type 1 |
|  | Proteins Involved in Diabetic Neuropathy |
|  | Immune Sustem Activation in Hashimoto's Thyroiditis |
|  | Hashimoto's Thyroiditis |
|  | Proteins Involved in Graves Disease |
|  | Graves Ophthalmopathy |
|  | Immune System Activation in Hyperthyroidism |
|  | Proteins Involved in Myasthenia Gravis |
|  | Proteins Involved in Obesity |
|  | Proteins Involved in Celiac Disease |
|  | CD4+T-Cell Response in Celiac Disease |
|  | Proteins with Altered Expression in Celiac Disease |
|  | Celiac Disease |
|  | B-Cell Activation in Crohn's Disease |
|  | Macrophage Dysfunction |
|  | Proteins Involved in Inflammatory Bowel Diseases |
|  | Proteins Involved in Helicobacter Infections |
|  | Leukocyte Migration toward the Endothelial Cell in Ulcerative Colitis |
|  | Proteins Involved in Ulcerative Colitis |
|  | Th2-Cell Function in Ulcerative Colitis |
|  | Proteins Involved in Diffuse Large-B-Cell Lymphoma |
|  | Follicular Lymphoma |
|  | Osteoclast Activation in Multiple Myeloma |
|  | Proteins Involved in Multiple Myeloma |
|  | Proteins Involved in Hepatocellular Carcinoma |
|  | ADAM33 Role in Asthma |
|  | Eosinophil Survival in Asthma |
|  | IgE Induces Airway Smooth Muscle Cell Proliferation |
|  | Goblet-Cell Exocytosis in Asthma |
|  | Goblet-Cell Related Mucus Secretion in Asthma |
|  | Club-Cell Role in Asthma |
|  | Basophil Activation in Asthma |
|  | Antigen-Presenting Cell Role in Asthma |
|  | B-Cell Mediated IgE Production in Asthma |
|  | Th2-Cell Response in Asthma |
|  | Mucin Production in Goblet Airway Epithelial Cells |
|  | Proteins Involved in Osteoarthritis |
|  | PTPN22 Role in Rheumatoid Arthritis |
|  | Proteins Involved in Rheumatoid Arthritis |
|  | FAS Mediated Apoptosis in Systemic Scleroderma |
|  | B-Cells Function in Systemic Scleroderma |
|  | Th2-Cells Function in Systemic Scleroderma |
|  | Proteins Involved in Systemic Scleroderma |
|  | Proteins with Altered Expression in Systemic Scleroderma |
|  | Proteins Involved in HIV Type 1 Infection |
|  | CD4+ T-Cell Function Decline in HIV |
|  | Proteins Involved in Periodontitis |
|  | Proteins Involved in Glioblastoma |
|  | Proteins Involved in Astrocytoma |
|  | Proteins Involved in Glioma |
|  | Proteins Involved in Neuroblastoma |
|  | MBP/MOG/PLP in Immune System Activation |
|  | Proteins Involved in Multiple Sclerosis |
|  | Proteins Involved in Endometriosis |
|  | Proteins Involved in Spontaneous Abortion |
|  | Genes with Mutations Associated with Asthma |
|  | Proteins Involved in Chronic Obstructive Pulmonary Disease |
|  | Mucin Hyperproduction in Goblet and Mucous Cells |
|  | Proteins Involved in Chronic Bronchitis |
|  | Proteins Involved in Polycystic Kidney Disease |
|  | Noradrenaline/Gs Expression Targets |
|  | Noreadrenaline/Gq Expression Targets |
|  | CD19 Expression Targets |
|  | CD81 -> AP-1 Expression Targets |
|  | CD8 -> AP-1 Expression Targets |
|  | CD72 -> CREB/CREBBP Expression Targets |
|  | CD72 -> STAT Expression Target |
|  | CD8 -> ATF/CREB/CREBBP Expression Targets |
|  | CD80 -> AP-1 Expression Targets |
|  | CD80 -> STAT Expression Targets |
|  | CD81 Expression Targets |
|  | CD80 -> NF-kB Expression Targets |
|  | CD86 -> STAT Expression Targets |
|  | CD86-> NFATC Expression Targets |
|  | CD86 -> NF-kB Expression Targets |
|  | CSF2 -> NF-kB Expression Targets |
|  | CSF2 -> STAT Expression Targets |
|  | Tumor Infiltrating Macrophages in Cancer Progression and Immune Escape |
|  | Myeloid Derived Suppressor Cells in Cancer Immune Escape |
|  | Proteins with Altered Expression in Cancer Immune Escape |
|  | Insulin -> STAT Expression Targets |
|  | Insulin -> CEBPA/CTNNB/FOXA/FOXO Expression Targets |
|  | Insulin -> MEF/MYOD Expression Targets |
|  | Insulin -> ELK/SRF/HIF1A/MYC/SREBF Expression Targets |
|  | IFNA1/IFNR Expression Targets |
|  | IFNG/IFNR Expression Targets |
|  | IL4R -> STAT Signaling |
|  | IL4R -> ELK/SRF/HMGY Signaling |
|  | IL7 Expression Targets |
|  | IL15 Expression Targets |
|  | IL12B Expression Targets |
|  | IL3 Expression Targets |
|  | IL4 Expression Targets |
|  | IL2 Expression Targets |
|  | PAF/Gq -> AP-1/ATF1/CREB/ERK/SRF Expression Targets |
|  | Morphine Expression Targets |
|  | PAF/Gq -> NF-kB Expression Targets |
|  | PGE1 Expression Targets |
|  | Proteins/Ions Transporters -> Expression Targets in Lymphoid System and Blood |
|  | Elevated Receptors -> Expression Targets in Lymphoid System and Blood |
|  | NOTCH -> Expression Targets in Thymus |
|  | Elevated Receptors -> Expression Targets in Thymus |
|  | NGF -> SMAD3/NF-kB Expression Targets |
|  | NGF -> AP-1/TP53/MYC Expression Targets |
|  | NGF -> CREB/CEBPB/MEF2A Expression Targets |
|  | JAG1 Expression Targets |
|  | NOTCH Expression Targets |
|  | Adenosine Expression Targets |
|  | GNRH1 Expression Targets |
|  | CCL11 Expression Targets |
|  | NPY Expression Targets |
|  | Collagen -> NF-kB Expression Targets |
|  | PAF Expression Targets |
|  | TCR -> NF-kB Expression Targets |
|  | TCR -> NFAT Expression Targets |
|  | TCR -> AP-1 Expression Targets |
|  | TGFB1-TGFBR1 Expression Targets |
|  | TNFSF14 Expression Targets |
|  | CD72 -> AP-1 Expression Targets |
|  | CD40LG -> NF-kB/ELK/SRF -> CREB/NFATC Expression Targets |
|  | TNFSF13B Expression Targets |
|  | TLR4 -> AP-1 Expression Targets |
|  | TLR4 -> NF-kB/IRF Expression Targets |
|  | B-Cell Differentiation Inhibition by Dioxin |
|  | PPAR Psoriasis |
|  | Model of PPARG signaling in psoriasis |
|  | IFN signaling in alveolar cells |
|  | Overview. Diseases of the skin (chapter 11) |
|  | Overview. Diseases of the respiratory (chapter 9) |
|  | 2\_Acute phase initiation in atopic dermatitis |
|  | 1\_Differentiation of psoriatic T cells |
|  | 1\_The onset of atopic dermatitis |
|  | 1\_Th2 cell response in asthma |
|  | 3\_2\_Regulation of airway surface liquid (ASL) thickness |
|  | 3\_1\_Mucus accumulation in asthma |
|  | 2\_2\_Eosinophilia and neutrophilia in asthma: eosinophil apoptosis |
|  | 4\_Mucin hyperproduction in goblet and mucous cells in COPD |
|  | 3\_Leukocyte migration toward the endothelial cells in the intestine microvasculature |
|  | 2\_Intestine epithelial cells drive immunological responses in ulcerative colitis |
|  | 4\_1\_Polymorphisms associated with inflammatory bowel diseases |
|  | 1\_CD4+ T cell and epithelial cell response in celiac disease |
|  | 2\_Defective tolerance of autoreactive T-cell in T1D |
|  | drug-target moltransport |
|  | Differentiation of psoriatic T cells |

---

|  |  |
| --- | --- |
| Group | Genes with Mutations Associated with Atopic Dermatitis |
|  | Genes with Mutations Associated with Psoriasis |
|  | Genes Associated with Systemic Lupus Erythematosus |
|  | Genes with Mutations Associated with Vitiligo |
|  | Genes with Mutations Associated with Hashimoto's Thyroiditis |
|  | Genes with Mutations Associated with Asthma |
|  | Genes with Mutations Associated with Multiple Sclerosis |

---

|  |  |
| --- | --- |
| Source | curated |
|  | GO component |

---

|  |  |
| --- | --- |
| MedScan ID | 3565 |

---

|  |  |
| --- | --- |
| LocusLink ID | 3565 |
|  | 16189 |
|  | 287287 |
|  | 24496 |
|  | 280824 |

---

|  |  |
| --- | --- |
| Alias | IGG1 induction factor |
|  | IL4R ligands |
|  | interleukin IL4\_HUMAN |
|  | B-cell IgG differentiation factor |
|  | BSF I |
|  | b cell stimulating factor |
|  | IL4e12 |
|  | interleukin IL4 |
|  | RP23-188H3.4 |
|  | IL4 |
|  | B cell stimulating factor 1 |
|  | IL IV |
|  | interleukin[4] |
|  | interleukin IV |
|  | BCGF-1 |
|  | b lymphocyte stimulating factor 1 |
|  | B-cell stimulatory factor 1 |
|  | interleukin(4) |
|  | interleukin IL4e12 |
|  | interleukin-4 receptor ligand |
|  | interleukin IL IV |
|  | interleukin 4 variant 2 |
|  | B\_cell stimulatory factor 1 |
|  | binetrakin |
|  | MGC79402 |
|  | BSF-1 |
|  | IL-4 receptor ligand |
|  | OTTMUSP00000005817 |
|  | Lymphocyte stimulatory factor 1 |
|  | B-cell stimulatory factor I |
|  | pitrakinra |
|  | B cell growth factor 1 |
|  | Lymphocyte stimulatory factor I |
|  | IL4\_HUMAN |
|  | BSF1 |
|  | IL-4 |
|  | BCGF1 |
|  | interleukin-4 |
|  | B-cell growth factor 1 |
|  | IL[4] |
|  | interleukin 4 |
|  | Interleukin-IL4 |
|  | IgG1 |

---

|  |  |
| --- | --- |
| GO ID | 0005125 |
|  | 0008083 |
|  | 0005136 |
|  | 0030183 |
|  | 0042110 |
|  | 0035745 |
|  | 0042976 |
|  | 0008203 |
|  | 0045189 |
|  | 0019221 |
|  | 0097028 |
|  | 0006955 |
|  | 0043011 |
|  | 0043066 |
|  | 1903660 |
|  | 2000352 |
|  | 0010633 |
|  | 0150079 |
|  | 0045671 |
|  | 0045892 |
|  | 0042536 |
|  | 0150076 |
|  | 2001171 |
|  | 0030890 |
|  | 0045348 |
|  | 0045582 |
|  | 0042102 |
|  | 1900223 |
|  | 1901857 |
|  | 0120162 |
|  | 0010628 |
|  | 0032736 |
|  | 0048295 |
|  | 0048304 |
|  | 0016239 |
|  | 0048260 |
|  | 0045944 |
|  | 0045893 |
|  | 0042531 |
|  | 0050776 |
|  | 0045191 |
|  | 0042325 |
|  | 0042092 |
|  | 0005576 |
|  | 0005615 |
|  | 0005126 |
|  | 0042113 |
|  | 0031296 |
|  | 0002296 |
|  | 0045064 |
|  | 0006914 |
|  | 0042832 |
|  | 0097192 |
|  | 0002227 |
|  | 0001774 |
|  | 0050868 |
|  | 2000320 |
|  | 0002674 |
|  | 0002677 |
|  | 2001237 |
|  | 0043031 |
|  | 0045019 |
|  | 0070351 |
|  | 0050871 |
|  | 0051091 |
|  | 0042104 |
|  | 0008284 |
|  | 0045080 |
|  | 0002230 |
|  | 2000424 |
|  | 0002639 |
|  | 0032733 |
|  | 0043306 |
|  | 0071677 |
|  | 1901741 |
|  | 0050731 |
|  | 0001934 |
|  | 1903428 |
|  | 0050727 |
|  | 0010155 |
|  | 0009897 |
|  | 0071288 |
|  | 0007565 |
|  | 0046427 |
|  | 1903142 |
|  | 0046825 |
|  | 0010469 |
|  | 0034097 |
|  | 0042493 |
|  | 0045471 |
|  | 0007584 |
|  | 0014070 |
|  | 0060041 |
|  | 0006954 |
|  | 0006968 |
|  | 0005515 |
|  | 0006935 |
|  | 0050863 |
|  | 0042523 |
|  | 0016563 |
|  | 0016564 |
|  | 0043193 |
|  | 0008283 |
|  | 0042087 |

---

|  |  |
| --- | --- |
| KEGG ID | rno:287287 |
|  | hsa:3565 |
|  | mmu:16189 |

---

|  |  |
| --- | --- |
| MedLine Reference | 12004164 |
|  | 11884473 |
|  | 15251393 |
|  | 15826818 |
|  | 16002150 |
|  | 16169702 |
|  | 16503976 |
|  | 16522370 |
|  | 16972262 |
|  | 17018025 |
|  | 1903705 |
|  | 3497723 |
|  | 10049944 |
|  | 10069422 |
|  | 10072503 |
|  | 10072509 |
|  | 10072551 |
|  | 10075870 |
|  | 10229108 |
|  | 10233740 |
|  | 10330435 |
|  | 10358179 |
|  | 10380772 |
|  | 10395689 |
|  | 10400816 |
|  | 10415077 |
|  | 10429671 |
|  | 10438933 |
|  | 10438949 |
|  | 10447769 |
|  | 10450518 |
|  | 10452966 |
|  | 10452977 |
|  | 10452990 |
|  | 10458752 |
|  | 10461958 |
|  | 10469236 |
|  | 10482018 |
|  | 10485657 |
|  | 10486156 |
|  | 10491413 |
|  | 10508526 |
|  | 10510085 |
|  | 10528202 |
|  | 10556817 |
|  | 10569763 |
|  | 10570276 |
|  | 10586035 |
|  | 10586118 |
|  | 10590269 |
|  | 10604990 |
|  | 10655497 |
|  | 10678951 |
|  | 10679097 |
|  | 10692110 |
|  | 10704283 |
|  | 10725799 |
|  | 10727445 |
|  | 10783149 |
|  | 10940898 |
|  | 10953030 |
|  | 10970823 |
|  | 10979977 |
|  | 10982767 |
|  | 10992460 |
|  | 11000402 |
|  | 11027433 |
|  | 11031101 |
|  | 11040185 |
|  | 11057672 |
|  | 11069076 |
|  | 11120785 |
|  | 11123351 |
|  | 11145720 |
|  | 11160238 |
|  | 11163225 |
|  | 11163865 |
|  | 11250124 |
|  | 11323701 |
|  | 11342667 |
|  | 11348452 |
|  | 11349033 |
|  | 11359854 |
|  | 11369703 |
|  | 11371356 |
|  | 11438376 |
|  | 11439159 |
|  | 11441125 |
|  | 11447288 |
|  | 11485928 |
|  | 11487546 |
|  | 11489976 |
|  | 11520464 |
|  | 11520798 |
|  | 11544284 |
|  | 11553589 |
|  | 11557514 |
|  | 11591797 |
|  | 11599050 |
|  | 11600887 |
|  | 11689671 |
|  | 11694459 |
|  | 11705919 |
|  | 11705956 |
|  | 11713466 |
|  | 11714822 |
|  | 11717189 |
|  | 11719504 |
|  | 11740499 |
|  | 11745342 |
|  | 11748180 |
|  | 11754358 |
|  | 11777945 |
|  | 11846461 |
|  | 11854255 |
|  | 11884290 |
|  | 11884434 |
|  | 11891315 |
|  | 11907061 |
|  | 11929777 |
|  | 11937535 |
|  | 11948403 |
|  | 11960636 |
|  | 1197194 |
|  | 11971948 |
|  | 11981811 |
|  | 11981831 |
|  | 11982753 |
|  | 11987243 |
|  | 12023955 |
|  | 12047751 |
|  | 12055229 |
|  | 12060463 |
|  | 12097365 |
|  | 12107190 |
|  | 12115603 |
|  | 12121972 |
|  | 12147624 |
|  | 12149469 |
|  | 12150887 |
|  | 12150890 |
|  | 12165489 |
|  | 12196290 |
|  | 12204227 |
|  | 12218118 |
|  | 12218156 |
|  | 12244147 |
|  | 12244163 |
|  | 12244176 |
|  | 12244202 |
|  | 12271139 |
|  | 12355446 |
|  | 12355448 |
|  | 12356576 |
|  | 12368275 |
|  | 12370375 |
|  | 12376365 |
|  | 12381344 |
|  | 12421669 |
|  | 12427968 |
|  | 12453468 |
|  | 12466851 |
|  | 12466896 |
|  | 12477932 |
|  | 12496409 |
|  | 12496449 |
|  | 12513913 |
|  | 12525563 |
|  | 12525570 |
|  | 12540559 |
|  | 12555667 |
|  | 12574343 |
|  | 12574379 |
|  | 12574393 |
|  | 12637316 |
|  | 12646610 |
|  | 12646613 |
|  | 12646651 |
|  | 12648490 |
|  | 12669034 |
|  | 12707364 |
|  | 12709397 |
|  | 12757264 |
|  | 12757709 |
|  | 12759427 |
|  | 12815549 |
|  | 12817750 |
|  | 12829028 |
|  | 12835475 |
|  | 12847246 |
|  | 12847266 |
|  | 12855407 |
|  | 12874364 |
|  | 12882831 |
|  | 12905482 |
|  | 12917264 |
|  | 12932361 |
|  | 12937153 |
|  | 12937416 |
|  | 12941137 |
|  | 12941478 |
|  | 12960353 |
|  | 1349581 |
|  | 1358285 |
|  | 1360278 |
|  | 1401920 |
|  | 1431102 |
|  | 1431136 |
|  | 14500669 |
|  | 14527170 |
|  | 14607954 |
|  | 14638820 |
|  | 14638848 |
|  | 14651591 |
|  | 14662870 |
|  | 14688111 |
|  | 14697238 |
|  | 14733929 |
|  | 14764670 |
|  | 14970176 |
|  | 14971043 |
|  | 14988498 |
|  | 14999688 |
|  | 15016857 |
|  | 15034018 |
|  | 15039362 |
|  | 15048712 |
|  | 15067040 |
|  | 15087305 |
|  | 15096484 |
|  | 15096489 |
|  | 15111500 |
|  | 15123681 |
|  | 15128777 |
|  | 15128834 |
|  | 15153506 |
|  | 15187136 |
|  | 15213137 |
|  | 15225294 |
|  | 15237109 |
|  | 15240659 |
|  | 15240669 |
|  | 15240692 |
|  | 15240716 |
|  | 15246867 |
|  | 15258154 |
|  | 15271961 |
|  | 15272080 |
|  | 15273769 |
|  | 1530915 |
|  | 15312135 |
|  | 15314695 |
|  | 15322148 |
|  | 15326095 |
|  | 15356151 |
|  | 15356173 |
|  | 15358865 |
|  | 15381187 |
|  | 15388256 |
|  | 1545131 |
|  | 15466391 |
|  | 15471863 |
|  | 15494498 |
|  | 15501755 |
|  | 15507491 |
|  | 15514019 |
|  | 15516924 |
|  | 15519344 |
|  | 15544617 |
|  | 15546395 |
|  | 15564479 |
|  | 15588696 |
|  | 15588938 |
|  | 15590890 |
|  | 1559701 |
|  | 15613333 |
|  | 15630137 |
|  | 15634756 |
|  | 15634927 |
|  | 15634931 |
|  | 15642955 |
|  | 15659391 |
|  | 15661875 |
|  | 15699131 |
|  | 15699158 |
|  | 15728505 |
|  | 15739164 |
|  | 15741272 |
|  | 15749878 |
|  | 15752732 |
|  | 15784551 |
|  | 15808517 |
|  | 15814686 |
|  | 15843535 |
|  | 15845902 |
|  | 1588041 |
|  | 15908341 |
|  | 15941711 |
|  | 15944265 |
|  | 15944273 |
|  | 1596564 |
|  | 15967820 |
|  | 15993493 |
|  | 15994233 |
|  | 16002690 |
|  | 16026997 |
|  | 16027141 |
|  | 16039582 |
|  | 16077007 |
|  | 16081830 |
|  | 16081840 |
|  | 16116164 |
|  | 16116211 |
|  | 16126843 |
|  | 1613462 |
|  | 16143305 |
|  | 16148098 |
|  | 16150647 |
|  | 16151533 |
|  | 16180247 |
|  | 16226507 |
|  | 16239566 |
|  | 16239576 |
|  | 16251181 |
|  | 16251408 |
|  | 16272308 |
|  | 16277637 |
|  | 16298365 |
|  | 16331625 |
|  | 16339553 |
|  | 16352630 |
|  | 16361318 |
|  | 16369024 |
|  | 16413168 |
|  | 16423063 |
|  | 16472593 |
|  | 16478801 |
|  | 1649220 |
|  | 16513385 |
|  | 16517715 |
|  | 16531090 |
|  | 16540525 |
|  | 16546101 |
|  | 16547273 |
|  | 16585579 |
|  | 16598817 |
|  | 16628252 |
|  | 16628253 |
|  | 16670768 |
|  | 16680145 |
|  | 16688680 |
|  | 16702603 |
|  | 16778988 |
|  | 16782026 |
|  | 16814598 |
|  | 16849470 |
|  | 16909433 |
|  | 16914507 |
|  | 16914747 |
|  | 16943384 |
|  | 16949316 |
|  | 16988272 |
|  | 17015711 |
|  | 17016559 |
|  | 17035062 |
|  | 17035345 |
|  | 17041596 |
|  | 17048986 |
|  | 17068285 |
|  | 17111344 |
|  | 17154265 |
|  | 1937872 |
|  | 1948049 |
|  | 1969921 |
|  | 2377456 |
|  | 2466396 |
|  | 2563351 |
|  | 2784591 |
|  | 2789124 |
|  | 2902144 |
|  | 3005865 |
|  | 3029676 |
|  | 3083412 |
|  | 3498301 |
|  | 6977612 |
|  | 7485382 |
|  | 7507352 |
|  | 7507968 |
|  | 7512032 |
|  | 7515717 |
|  | 7515912 |
|  | 7517363 |
|  | 7532600 |
|  | 7591152 |
|  | 7594565 |
|  | 7598937 |
|  | 7609051 |
|  | 7613115 |
|  | 7636230 |
|  | 7691937 |
|  | 7691943 |
|  | 7705393 |
|  | 7720090 |
|  | 7729546 |
|  | 7750457 |
|  | 7760829 |
|  | 7790826 |
|  | 7807002 |
|  | 7807021 |
|  | 7826951 |
|  | 7873190 |
|  | 7874121 |
|  | 7903095 |
|  | 7927664 |
|  | 7929391 |
|  | 7937857 |
|  | 7974717 |
|  | 7996051 |
|  | 8012109 |
|  | 8034712 |
|  | 8035532 |
|  | 8045595 |
|  | 8051417 |
|  | 8052653 |
|  | 8088835 |
|  | 8093672 |
|  | 8100556 |
|  | 8133052 |
|  | 8145047 |
|  | 8155595 |
|  | 8188659 |
|  | 8200040 |
|  | 8207122 |
|  | 8211142 |
|  | 8240343 |
|  | 8266076 |
|  | 8313907 |
|  | 8376797 |
|  | 8384701 |
|  | 8406478 |
|  | 8444353 |
|  | 8496684 |
|  | 8524877 |
|  | 8551213 |
|  | 8584936 |
|  | 8595891 |
|  | 8602263 |
|  | 8605347 |
|  | 8612134 |
|  | 8617946 |
|  | 8624821 |
|  | 8626013 |
|  | 8661733 |
|  | 8666240 |
|  | 8742061 |
|  | 8754800 |
|  | 8755560 |
|  | 8764032 |
|  | 8765046 |
|  | 8770556 |
|  | 8799723 |
|  | 8814247 |
|  | 8833033 |
|  | 8834765 |
|  | 8840532 |
|  | 8875898 |
|  | 8906833 |
|  | 8911153 |
|  | 8916957 |
|  | 8921430 |
|  | 8934515 |
|  | 8943375 |
|  | 8943570 |
|  | 8977198 |
|  | 8977282 |
|  | 8992981 |
|  | 8992993 |
|  | 8996242 |
|  | 8999817 |
|  | 9000540 |
|  | 9013979 |
|  | 9064329 |
|  | 9070927 |
|  | 9109438 |
|  | 9110977 |
|  | 9122622 |
|  | 9123210 |
|  | 9130646 |
|  | 9133425 |
|  | 9133426 |
|  | 9137108 |
|  | 9162077 |
|  | 9175832 |
|  | 9175833 |
|  | 9177770 |
|  | 9184926 |
|  | 9199462 |
|  | 9257845 |
|  | 9310836 |
|  | 9317156 |
|  | 9344502 |
|  | 9366429 |
|  | 9366559 |
|  | 9379007 |
|  | 9382883 |
|  | 9422330 |
|  | 9449711 |
|  | 9482907 |
|  | 9492006 |
|  | 9498745 |
|  | 9500796 |
|  | 9501881 |
|  | 9504339 |
|  | 9531306 |
|  | 9538130 |
|  | 9548487 |
|  | 9551937 |
|  | 9551967 |
|  | 9551990 |
|  | 9558109 |
|  | 9565640 |
|  | 9590229 |
|  | 9590248 |
|  | 9590259 |
|  | 9619371 |
|  | 9625018 |
|  | 9635196 |
|  | 9636366 |
|  | 9670859 |
|  | 9670967 |
|  | 9680378 |
|  | 9686617 |
|  | 9703318 |
|  | 9712058 |
|  | 9729042 |
|  | 9732697 |
|  | 9754555 |
|  | 9759845 |
|  | 9759875 |
|  | 9780277 |
|  | 9802888 |
|  | 9820512 |
|  | 9824514 |
|  | 9858522 |
|  | 9862708 |
|  | 9878080 |
|  | 9881967 |
|  | 9885919 |
|  | 9887201 |
|  | 9893036 |
|  | 9916075 |
|  | 9922312 |
|  | 9922399 |
|  | 9933080 |
|  | 9973388 |
|  | 10438901 |
|  | 10473513 |
|  | 10725748 |
|  | 10753117 |
|  | 11149986 |
|  | 11320251 |
|  | 11396683 |
|  | 11418631 |
|  | 11466338 |
|  | 11587989 |
|  | 11676128 |
|  | 11728464 |
|  | 11777980 |
|  | 11786020 |
|  | 11811777 |
|  | 11841848 |
|  | 11842291 |
|  | 11872954 |
|  | 11886168 |
|  | 11897999 |
|  | 11907068 |
|  | 11920312 |
|  | 11967989 |
|  | 11968047 |
|  | 11994288 |
|  | 12039414 |
|  | 12063527 |
|  | 12066847 |
|  | 12072494 |
|  | 12102661 |
|  | 12105273 |
|  | 12107748 |
|  | 12115161 |
|  | 12133437 |
|  | 12144536 |
|  | 12161424 |
|  | 12162874 |
|  | 12165514 |
|  | 12207324 |
|  | 12220533 |
|  | 12366695 |
|  | 12402189 |
|  | 12426308 |
|  | 12435565 |
|  | 12441140 |
|  | 12470598 |
|  | 12479817 |
|  | 12504082 |
|  | 12508786 |
|  | 12509382 |
|  | 12513914 |
|  | 12515722 |
|  | 12517954 |
|  | 12525574 |
|  | 12542496 |
|  | 12552499 |
|  | 12574355 |
|  | 12587822 |
|  | 12600829 |
|  | 12613995 |
|  | 12626590 |
|  | 12660931 |
|  | 12663328 |
|  | 12689929 |
|  | 12738651 |
|  | 12748907 |
|  | 12813360 |
|  | 12851716 |
|  | 12877559 |
|  | 12884288 |
|  | 12897746 |
|  | 12902508 |
|  | 12940513 |
|  | 12944981 |
|  | 14499500 |
|  | 14503851 |
|  | 14530362 |
|  | 14561191 |
|  | 14568946 |
|  | 14583474 |
|  | 14610476 |
|  | 14634065 |
|  | 14634100 |
|  | 14652008 |
|  | 14654080 |
|  | 14681304 |
|  | 14688371 |
|  | 14692664 |
|  | 14697936 |
|  | 14725568 |
|  | 14729510 |
|  | 14746805 |
|  | 14764715 |
|  | 14767810 |
|  | 14983029 |
|  | 15001290 |
|  | 15004182 |
|  | 15007352 |
|  | 15013759 |
|  | 15014952 |
|  | 15041089 |
|  | 15042002 |
|  | 15051937 |
|  | 15084933 |
|  | 15100288 |
|  | 15122773 |
|  | 15128803 |
|  | 15163899 |
|  | 15166131 |
|  | 15187156 |
|  | 15210741 |
|  | 15213252 |
|  | 15274656 |
|  | 15297269 |
|  | 15322832 |
|  | 15367434 |
|  | 15474066 |
|  | 15501397 |
|  | 15527169 |
|  | 15528258 |
|  | 15570643 |
|  | 15591113 |
|  | 15627726 |
|  | 15676220 |
|  | 15678501 |
|  | 15695802 |
|  | 15714203 |
|  | 15778356 |
|  | 15816837 |
|  | 15825027 |
|  | 15869882 |
|  | 15883744 |
|  | 15885323 |
|  | 15900573 |
|  | 15919378 |
|  | 15951665 |
|  | 15969687 |
|  | 16001979 |
|  | 16004996 |
|  | 16034134 |
|  | 16044136 |
|  | 16190985 |
|  | 16215326 |
|  | 16249002 |
|  | 16343431 |
|  | 16388492 |
|  | 16427155 |
|  | 16461126 |
|  | 16466711 |
|  | 16492782 |
|  | 16551465 |
|  | 16574063 |
|  | 16596275 |
|  | 16601843 |
|  | 16640778 |
|  | 16707627 |
|  | 16750993 |
|  | 16810739 |
|  | 16864907 |
|  | 16877367 |
|  | 16930576 |
|  | 16931887 |
|  | 16987060 |
|  | 17006724 |
|  | 17015714 |
|  | 17015735 |
|  | 17063753 |
|  | 17097641 |
|  | 17141086 |
|  | 17148075 |
|  | 1993171 |
|  | 2535858 |
|  | 3016727 |
|  | 7695626 |
|  | 7806280 |
|  | 7963560 |
|  | 8266078 |
|  | 8516290 |
|  | 8603435 |
|  | 9113989 |
|  | 9130521 |
|  | 9130652 |
|  | 9334723 |
|  | 9856950 |
|  | 1601309 |
|  | 7626886 |
|  | 10219247 |
|  | 1400355 |
|  | 1511746 |
|  | 15489334 |
|  | 1567880 |
|  | 1567881 |
|  | 1569578 |
|  | 1609277 |
|  | 16690518 |
|  | 16973957 |
|  | 17117487 |
|  | 17143971 |
|  | 17203290 |
|  | 17207024 |
|  | 17237413 |
|  | 17299718 |
|  | 17309781 |
|  | 17371990 |
|  | 17379175 |
|  | 17389011 |
|  | 17389870 |
|  | 17433443 |
|  | 17451413 |
|  | 17460561 |
|  | 17541280 |
|  | 17548690 |
|  | 17566759 |
|  | 17631734 |
|  | 17644317 |
|  | 1932028 |
|  | 1946344 |
|  | 3257560 |
|  | 7664036 |
|  | 8151703 |
|  | 10207161 |
|  | 10209035 |
|  | 10229862 |
|  | 10558953 |
|  | 10843696 |
|  | 10861073 |
|  | 10878373 |
|  | 10878379 |
|  | 10973285 |
|  | 11034364 |
|  | 11034365 |
|  | 11714756 |
|  | 12218103 |
|  | 12218136 |
|  | 12444136 |
|  | 12471132 |
|  | 12507769 |
|  | 12642601 |
|  | 12695491 |
|  | 16020287 |
|  | 16210602 |
|  | 16210629 |
|  | 16210636 |
|  | 16237054 |
|  | 16272288 |
|  | 16275384 |
|  | 16301612 |
|  | 16339525 |
|  | 16606668 |
|  | 16769892 |
|  | 16880257 |
|  | 17164429 |
|  | 17182558 |
|  | 17195845 |
|  | 17200144 |
|  | 17215437 |
|  | 17237387 |
|  | 17241396 |
|  | 17244480 |
|  | 17277164 |
|  | 17304625 |
|  | 17304630 |
|  | 17318261 |
|  | 17318894 |
|  | 17325050 |
|  | 17330244 |
|  | 17339609 |
|  | 17412309 |
|  | 17450126 |
|  | 17476359 |
|  | 17487773 |
|  | 17513694 |
|  | 17532053 |
|  | 17579059 |
|  | 17601518 |
|  | 17611223 |
|  | 17646405 |
|  | 17670977 |
|  | 7873877 |
|  | 8022486 |
|  | 9463404 |
|  | 9637480 |
|  | 9647229 |
|  | 9670933 |
|  | 9743346 |
|  | 9811885 |
|  | 17442043 |
|  | 1983334 |

---

|  |  |
| --- | --- |
| Organism | Homo sapiens {Organism urn:agi-taxid:9606} |
|  | Mus musculus {Organism urn:agi-taxid:10090} |
|  | Rattus norvegicus {Organism urn:agi-taxid:10116} |
|  | Homo sapiens |
|  | Mus musculus |
|  | Rattus norvegicus |
|  | Bos taurus |

---

|  |  |
| --- | --- |
| Mouse chromosome position | 11 31.97 cM |
|  | 11 29.0 cM |

---

|  |  |
| --- | --- |
| OMIM ID | 147780 |

---

|  |  |
| --- | --- |
| Rat chromosome position | 10q22 |

---

|  |  |
| --- | --- |
| Hugo ID | 6014 |
|  | HGNC:6014 |

---

|  |  |
| --- | --- |
| Human chromosome position | 5q31.1 |

---

|  |  |
| --- | --- |
| Swiss-Prot Accession | D4HNR6 |
|  | P05112 |
|  | Q5FC01 |
|  | P05112.1 |
|  | P07750 |
|  | P07750.1 |
|  | P20096 |
|  | P20096.2 |
|  | Q14630 |
|  | Q6NZ77 |
|  | Q5SV00 |
|  | Q6NWP0 |
|  | Q9UPB9 |
|  | Q5KT56 |
|  | Q91Y50 |
|  | Q6RIC4 |

---

|  |  |
| --- | --- |
| PIR ID | A60237 |
|  | A30546 |
|  | A25870 |

---

|  |  |
| --- | --- |
| GenBank ID | NC\_000005 |
|  | NM\_001354990 |
|  | NP\_001341919 |
|  | NM\_000589 |
|  | NP\_000580 |
|  | NM\_172348 |
|  | NP\_758858 |
|  | NG\_023252 |
|  | AC004039 |
|  | AF395008 |
|  | AAK71324 |
|  | AF465829 |
|  | CH471062 |
|  | EAW62322 |
|  | EAW62323 |
|  | CS255762 |
|  | CAJ75955 |
|  | FB507239 |
|  | CAV32874 |
|  | FJ807883 |
|  | ADD60204 |
|  | GQ892002 |
|  | ADN03408 |
|  | GQ892003 |
|  | ADN03409 |
|  | GQ892004 |
|  | ADN03410 |
|  | GQ892005 |
|  | ADN03411 |
|  | GQ892006 |
|  | ADN03412 |
|  | GQ892007 |
|  | ADN03413 |
|  | GQ892008 |
|  | ADN03414 |
|  | GQ892009 |
|  | ADN03415 |
|  | GQ892010 |
|  | ADN03416 |
|  | GQ892011 |
|  | ADN03417 |
|  | GQ892012 |
|  | ADN03418 |
|  | GQ892013 |
|  | ADN03419 |
|  | GQ892014 |
|  | ADN03420 |
|  | GQ892015 |
|  | ADN03421 |
|  | GQ892016 |
|  | ADN03422 |
|  | GQ892017 |
|  | ADN03423 |
|  | HC679221 |
|  | CBL86566 |
|  | HH829894 |
|  | CBX32734 |
|  | M23442 |
|  | AAA59150 |
|  | X06750 |
|  | CAA29925 |
|  | AB102862 |
|  | BAD89396 |
|  | AF043336 |
|  | AAC03537 |
|  | AM937235 |
|  | CAP72493 |
|  | BC066277 |
|  | AAH66277 |
|  | BC066278 |
|  | BC067514 |
|  | AAH67514 |
|  | BC067515 |
|  | AAH67515 |
|  | BC070123 |
|  | AAH70123 |
|  | CD640452 |
|  | EF173851 |
|  | ABM53124 |
|  | JX878389 |
|  | AGM38212 |
|  | M13982 |
|  | AAA59149 |
|  | X81851 |
|  | CAA57444 |
|  | P05112 |
|  | NC\_000077 |
|  | NM\_021283 |
|  | NP\_067258 |
|  | NR\_027491 |
|  | AC005742 |
|  | AL596095 |
|  | AL645741 |
|  | CH466575 |
|  | EDL33576 |
|  | EDL33577 |
|  | EDL33578 |
|  | U07869 |
|  | X05064 |
|  | CAA28731 |
|  | X05252 |
|  | CAA28873 |
|  | X05253 |
|  | CAA28874 |
|  | AA199406 |
|  | AB174765 |
|  | BAD83771 |
|  | AF352783 |
|  | AAK52901 |
|  | AF359558 |
|  | BC027514 |
|  | AAH27514 |
|  | BX529138 |
|  | M13238 |
|  | AAA39307 |
|  | M25892 |
|  | AAA39298 |
|  | X03532 |
|  | CAA27233 |
|  | P07750 |
|  | NC\_005109 |
|  | NM\_201270 |
|  | NP\_958427 |
|  | AC\_000078 |
|  | AAHX01063900 |
|  | AAHX01063901 |
|  | AC107611 |
|  | CH473948 |
|  | EDM04390 |
|  | EDM04391 |
|  | EDM04392 |
|  | EDM04393 |
|  | X53087 |
|  | CAA37256 |
|  | X53088 |
|  | X53089 |
|  | AY496861 |
|  | AAR87867 |
|  | X16058 |
|  | CAC16091 |
|  | P20096 |
|  | CAI24351 |
|  | AAHY01093363 |
|  | AMYH02012219 |
|  | AC\_000033 |
|  | AC\_000137 |
|  | AABR06063818 |
|  | CAI24688 |
|  | ABBA01068803 |
|  | XR\_427710 |
|  | NC\_018916 |
|  | NT\_034772 |
|  | NW\_922784 |
|  | NW\_001838952 |
|  | AC\_000048 |
|  | Q5FC01 |
|  | Q6NWP0 |
|  | Q6NZ77 |
|  | Q9UPB9 |
|  | NT\_096135 |
|  | NW\_001030461 |
|  | Q5KT56 |
|  | Q5SV00 |
|  | Q91Y50 |
|  | NW\_047334 |
|  | NW\_001084656 |
|  | Q6RIC4 |
|  | NT\_039530 |
|  | AAH66278 |
|  | NW\_000039 |
|  | 11140161 |
|  | 124339 |
|  | 40557191 |
|  | 4894184 |
|  | 81864133 |
|  | 124338 |
|  | 1334110 |
|  | 1334111 |
|  | 14030446 |
|  | 20380614 |
|  | 309412 |
|  | 51827 |
|  | 52679 |
|  | 533237 |
|  | 56205961 |
|  | 56206963 |
|  | 57157562 |
|  | 81889114 |
|  | 81902427 |
|  | 81910145 |
|  | 119582726 |
|  | 119582727 |
|  | 124337 |
|  | 14600280 |
|  | 186337 |
|  | 2905624 |
|  | 307061 |
|  | 33832 |
|  | 42490871 |
|  | 42490900 |
|  | 45708996 |
|  | 45709848 |
|  | 47123367 |
|  | 58736975 |
|  | 673419 |
|  | 74721228 |
|  | 74721688 |
|  | 74736926 |
|  | 74737031 |
|  | 86476574 |
|  | NT\_086681 |
|  | 163211 |
|  | 266339 |
|  | 555896 |
|  | AAA30587 |
|  | AAA82730 |
|  | M77120 |
|  | NC\_007305 |
|  | NM\_173921 |
|  | NP\_776346 |
|  | NW\_001495335 |
|  | P30367 |
|  | U14159 |
|  | U14160 |
|  | 148701629 |
|  | 148701630 |
|  | 148701631 |
|  | 149052573 |
|  | 149052574 |
|  | 149052575 |
|  | 149052576 |

---

|  |  |
| --- | --- |
| Swiss-Prot ID | IL4\_RAT |
|  | IL4\_HUMAN |
|  | IL4\_MOUSE |
|  | Q5FC01\_HUMAN |
|  | D4HNR6\_HUMAN |
|  | Q5SV00\_MOUSE |

---

|  |  |
| --- | --- |
| Cell Localization | Secreted |
|  | Extracellular |

---

|  |  |
| --- | --- |
| Ensembl ID | ENSG00000113520 |
|  | ENSP00000231449.2 |
|  | ENST00000231449.7 |
|  | ENSP00000480581.1 |
|  | ENST00000622422.1 |
|  | ENSP00000325190.3 |
|  | ENST00000350025.2 |
|  | ENSMUSG00000000869 |
|  | ENSMUSP00000000889.6 |
|  | ENSMUST00000000889.6 |
|  | ENSMUST00000150568.7 |
|  | ENSRNOG00000007624 |
|  | ENSRNOP00000010029.1 |
|  | ENSRNOT00000010029.3 |
|  | ENST00000231449.6 |
|  | ENSMUSP00000000889 |
|  | ENSP00000325190 |
|  | ENSMUST00000150568 |
|  | ENSRNOP00000010029 |
|  | ENSRNOT00000010029 |
|  | ENST00000350025 |
|  | ENSMUST00000000889 |
|  | ENST00000231449 |
|  | ENSP00000231449 |

---

|  |  |
| --- | --- |
| MGI ID | MGI:96556 |
|  | 96556 |

---

|  |  |
| --- | --- |
| RGD ID | 2898 |

---

|  |  |
| --- | --- |
| Unigene ID | Mm.276360 |
|  | Rn.108255 |
|  | Hs.73917 |
|  | Bt.423 |

---

|  |  |
| --- | --- |
| Homologene ID | 491 |

---

|  |  |
| --- | --- |
| Shape | Rhomb |

---

|  |  |
| --- | --- |
| IPI ID | IPI00217945 |
|  | IPI00131815 |
|  | IPI00007081 |
|  | IPI00195650 |
|  | IPI00454514 |

---

|  |  |
| --- | --- |
| Microarray ID | 92283\_s\_at |
|  | 92284\_r\_at |
|  | 92285\_at |
|  | 92286\_g\_at |
|  | AFFX-MurIL4\_at |

---

|  |  |
| --- | --- |
| Homo sapiens Chromosome position | 5q31.1 |

---

|  |  |
| --- | --- |
| Ensembl Transcript ID | ENST00000350025 |
|  | ENST00000231449 |

---

|  |  |
| --- | --- |
| FunctionalClass | cytokine |
|  | interleukin-4 receptor binding |
|  | protein binding |
|  | growth factor |
|  | hematopoietin/interferon-class (D200-domain) cytokine receptor binding |

---

|  |  |
| --- | --- |
| Bos taurus Chromosome position | 7q15-q21 |

---

|  |  |
| --- | --- |
| Mus musculus Chromosome position | 11 29.0 cM |

---

|  |  |
| --- | --- |
| KEGG pathway | Hematopoietic cell lineage |
|  | Cytokine-cytokine receptor interaction |
|  | Jak-STAT signaling pathway |
|  | T cell receptor signaling pathway |
|  | Fc epsilon RI signaling pathway |

---

|  |  |
| --- | --- |
| Rattus norvegicus Chromosome position | 10 |
|  | 10q22 |

---

|  |  |
| --- | --- |
| Ensembl Gene ID | ENSG00000113520 |

---
